# New therapies for spinal muscular atrophy: where we stand and what is next

**DOI:** 10.1007/s00431-023-04883-8

**Published:** 2023-04-17

**Authors:** Laura Antonaci, Maria Carmela Pera, Eugenio Mercuri

**Affiliations:** 1 Centro Clinico Nemo, Fondazione Policlinico Universitario Agostino Gemelli IRCCS, Rome, Italy; 2 Pediatric Neurology, Università Cattolica del Sacro Cuore, Rome, Italy

**Keywords:** Spinal muscular atrophy, Therapy, Clinical trial, Real-world data, Combined therapies

## Abstract

The natural history of spinal muscular atrophy has been radically changed by the advent of improved standards of care and the availability of disease-modifying therapies. The aim of this paper is to provide the current therapeutic scenario including new perspectives and to report the challenges related to new phenotypes a few years after the therapies have become available. The paper also includes a review of real-world data that provides information on safety and efficacy in individuals that were not included in clinical trials. Special attention is paid to future perspectives both in terms of new drugs that are currently investigated in clinical trials or providing details on current developments in the use of the available drugs, including combination therapies or new modalities of dose or administration.

*  Conclusion*: Clinical trials and real world data support the efficacy and safety profiles of the available drugs. At the moment there is not enough published evidence about the superiority of one product compared to the others.

**Table:** 

**What is Known:**
• *Safety and efficacy results of clinical trials have led in the last 6 years to the marketing of three drugs for spinal muscular atrophy, with different mechanisms of action.*
**What is New:**
• *Since the drug’s approval, real-world data allow us to have data on bigger and heterogeneous groups of patients in contrast with those included in clinical trials.*
•* In addition to the new molecules, combinations of therapies are currently being evaluated.*

## Introduction

Spinal muscular atrophy (SMA) is a genetic disease characterized by the degeneration of α-motor neurons of the anterior horns of the spinal cord resulting in progressive muscle weakness [1]. Approximately 95% of patients with 5q-SMA show homozygous deletions of either exons 7 and 8 or only exon 7 of *SMN1* that is responsible for the expression of most of the functional SMN proteins.

Historically, the classification of SMA was based on the age of onset of symptoms and maximum motor acquisition [2–6]. SMA type I, the most severe type, is characterized by onset before 6 months, with inability to reach sitting position and had a reduced life expectancy. In type II, the symptom onset is between 6 and 18 months of age. Patients never acquired independent walking [6]. In type III, the onset is after 18 months, and in a number of cases, patients lose the ability to walk [7]. This classification has become obsolete as, due to the advent of the new therapies, there has been a dramatic change in survival, in maximum motor function achieved, and in the overall progression of the disease [6].

In this review, we will describe the state of art of the currently available therapeutic approaches, reviewing clinical trials and real-world data, focusing on the impact of the new therapies on the “new” natural history and on the possible next steps in the field.

## Available therapeutical approaches

**Fig. 1:**
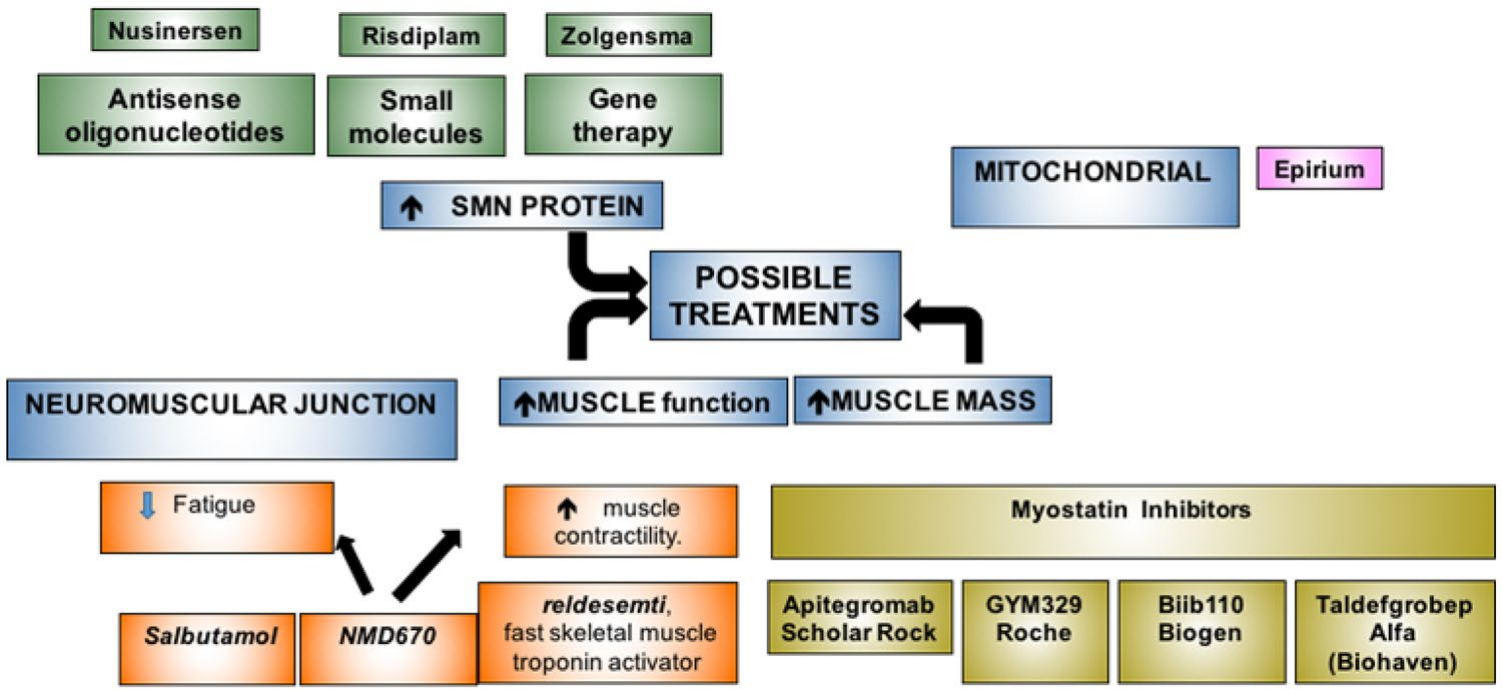
Summary of the main approaches subdivided according to their mechanism of action

Different studies have been targeting different steps of the pathogenetic mechanism, from the replacement of the affected gene to approaches targeting the motoneurons or, peripherally, muscle or neuromuscular junction (Fig. 1).

## Increasing SMN protein

**Fig. 2:**
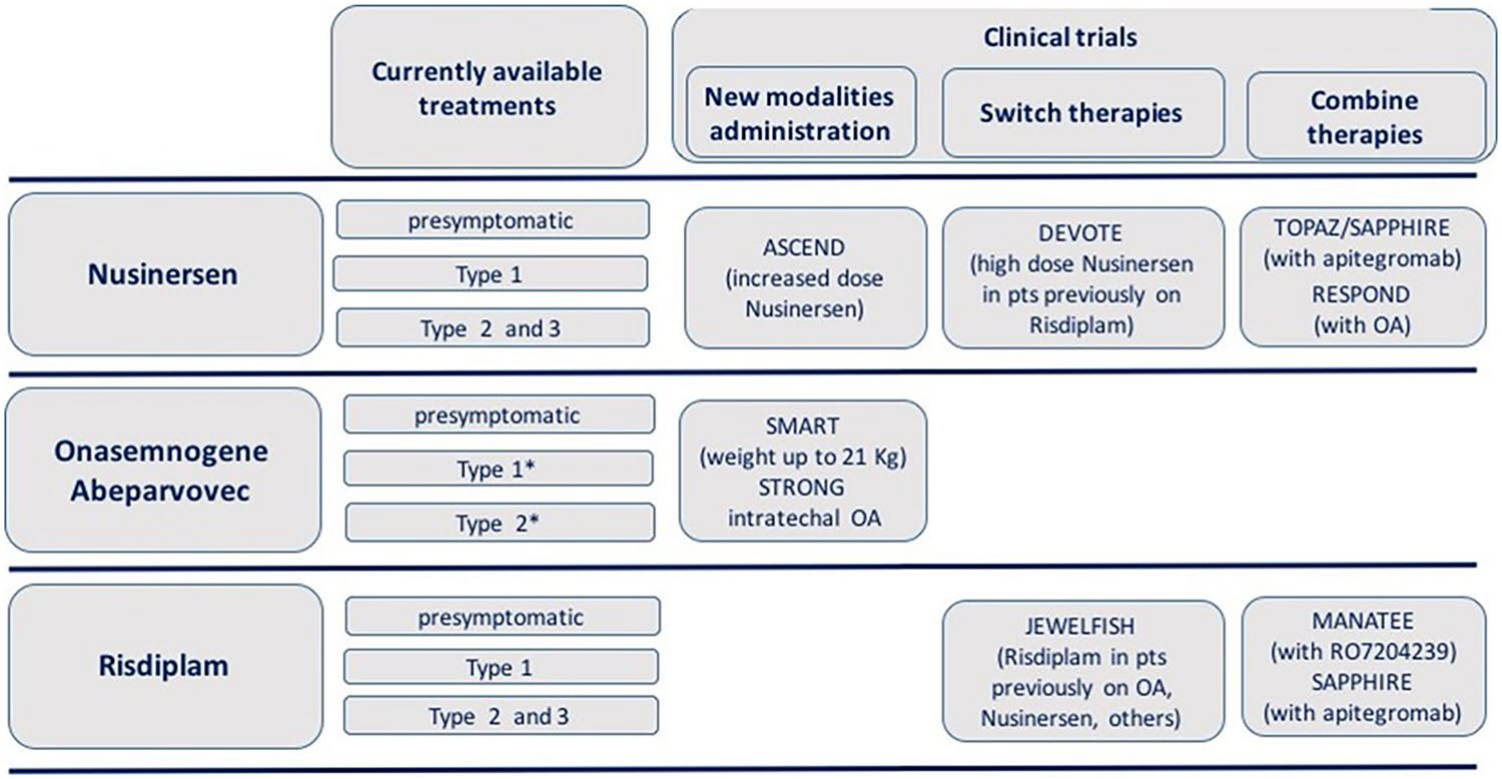
Overview of the currently available treatment and of the ongoing developments

The three therapies that so far have been approved are all targeting an increase of the protein survival motor neuron protein (SMN) that is deficient in SMA. The two main mechanisms to increase SMN protein are related to *SMN1* gene replacement or to target SMN2 splicing at mRNA level [7, 8]. Figure 2 provides an overview of the currently available treatment and of the ongoing developments.

### Gene replacement: onasemnogene abeparvovec

This approach aims at addressing the root problem of the disease by replacing the mutated SMN1 gene [9]. The relatively small size of the SMN1 gene is compatible with the use of an adeno-associated viral vector (AAV9) that can cross the blood brain barrier. The main advantage of this approach is that a one-time intravenous injection will result in a systemic expression of the *SMN* protein. Possible disadvantages include lack of long-term efficacy and safety data.

#### Onasemnogene abeparvovec: clinical trials

Onasemnogene abeparvovec was first investigated in an open-label, dose-escalation phase I clinical trial (START NCT02122952) performed in 15 infants with early-onset SMA [9]. The drug was overall well tolerated. All survived beyond the age of 20 months, at the age when, in the absence of treatment, natural history studies reported a survival below 8% [3, 4]. Most children achieved the ability to sit and showed an improvement on the functional scale. The extension 5-year follow-up study showed that all patients were still alive and motor milestones were maintained or further improved [10].

Safety and efficacy data were confirmed by two large open-label, multicenter phase III studies (NCT02456740) in the USA and Europe in infants with early onset with an age below 7 months [11, 12]. The most common adverse events in the clinical trials were related to reduced platelets and elevated serum levels of aminotransferase.

#### Onasemnogene abeparvovec: real-world data

The number of studies reporting real-world data using onasemnogene abeparvovec is still limited. The approval of onasemnogene abeparvovec was granted with a wider label allowing to use the drug in infants till the age of 2 years (e.g., US) or up to a weight of 21 kg (Europe) [13]. Since the approval of the OA, several studies have reported safety and efficacy real-world data also including older and heavier children than those reported in clinical trials [14–17]. The studies confirm the efficacy observed in clinical trials, even in children who were older than 7 months at the time of treatment [14–17].

The real-world data suggest that elevated serum levels of aminotransferase and reduced platelets were more frequent in heavier infants but were always well controlled by prednisolone [14–20]. A new concern came from the observation of thrombotic microangiopathy (TMA) which had been reported in preclinical studies but had never been observed in the clinical trials [21]. The occurrence of TMA is a major concern but is very rare.

#### Onasemnogene abeparvovec: new developments

The published data reflect the labels provided in individual countries at the time of approval. Open-label multicenter studies are in progress to assess the safety of the biodistribution, safety, and tolerability of intravenous administration of high doses of AAV vector in children up to 21 kg in weight (SMART; NCT04851873) and to establish safety and efficacy of intrathecal AVXS-101 delivery (STRONG; NCT03381729).

### SMN2- and SMN2 transcript-directed therapies

The other approaches aiming at increasing SMN protein use a different mechanism, targeting not the SMN1 gene but the homologous gene SMN2. All SMA patients have mutations in the SMN1 gene but have at least one copy of SMN2. Each copy of SMN2 mainly produces rapidly degraded SMN protein and only a minimal part (~ 10%) of full-length, functional SMN protein. Altering the splicing of exon 7 of the SMN2 gene leads to a greater production of stable and more functional SMN protein. Two therapies have so far been approved, using antisense oligonucleotides or small molecules.

#### Nusinersen

Nusinersen, an antisense oligonucleotide, was the first drug to demonstrate that altering the splicing of the SMN2 pre-mRNA resulted in increased production of full-length SMN protein and in clinical efficacy [22–24].

##### Nusinersen: clinical trials

After promising results for nusinersen in phase I and II trials [25, 26], two clinical phase III, randomized, double-blind, sham-procedure controlled studies allowed the approval of this drug: ENDEAR (NCT02193074), which included SMA type I patients [23] and CHERISH (NCT02292537), for SMA late-onset patients [24], assessing safety and clinical efficacy.

The ENDEAR clinical trial in early-onset SMA showed a significant increase in event-free survival (defined as the time to death or the use of permanent assisted ventilation) in nusinersen-treated infants and an improvement in motor milestones versus sham control infants.

The results of the CHERISH trial showed a significant increase in the Hammersmith Functional Motor Scale–Expanded (HFMSE) score and in the Revised Upper Limb Module (RULM) scores.

Adverse event was mainly linked to the disease and to the intrathecal administration.

The efficacy data as well as long-term safety were confirmed by the open-label extension SHINE (NCT02594124) study confirming durability of the responses over time.

##### Nusinersen: real-world data

Real-world data collected after the approval of nusinersen or as part of early open-access programs [27–33] have confirmed safety and efficacy data in a much wider range of age, SMA type, age at treatment, and functional level than those used in clinical trials.

Several studies have confirmed increased survival and improved function in type I infants [27, 28, 30–32]. This was most obvious in the infants treated before the age of 6 months, but significant changes could also be observed in those treated within the first year, with smaller changes that could be observed in patients treated at an older age. We recently reported 24-month real-world data using nusinersen in infantile-onset SMA, showing some improvement of motor function can be observed even after the first year of treatment [34].

A number of other studies have also recently reported efficacy and safety of nusinersen in type II and III patients, also including ambulant and adult patients that had not been included in the pivotal clinical trials [29, 34–44]. A recent meta-analysis on motor function in type II and III patients treated with nusinersen including all the real-world data available in the literature [45] shows that in all studies nusinersen treatment was associated with a favorable benefit in motor function that could be observed even when subdividing the results according to age and type of assessment. These results were different from those observed in natural history untreated cohorts who consistently showed negative changes [34, 46].

##### New developments

An ongoing phase 2/3 study, DEVOTE (NCT04089566), is currently exploring efficacy, safety, and PK of higher doses of nusinersen. The pharmacokinetic/pharmacodynamic (PK/PD) analysis indicates that increased exposures obtained with a higher dose of nusinersen may lead to an increase in efficacy above that seen with the 12-mg approved dose.

A second study, ASCEND (NCT05067790), is a single-arm, open-label phase 3b study aimed to evaluate higher-dose nusinersen (BIIB058) in SMA patients previously treated with risdiplam.

Other studies currently exploring the combination of nusinersen with other drugs will be discussed in a separate section.

#### Risdiplam

Risdiplam is a small molecule that, like nusinersen, modifies SMN2 pre-mRNA splicing. This small molecule crosses the blood brain barrier and is administered orally once daily reaching bioavailability in both central and peripheral tissues [47].

##### Risdiplam: clinical trials

Following phase I trials [48], two pivotal studies were performed in early and late-onset SMA. FIREFISH (NCT02913482) was a multicenter, open-label, two-part study using risdiplam in infants with early infantile SMA [49, 50]. The study assessed the efficacy and safety of risdiplam in infants aged 1 to 7 months, with two *SMN2* gene copies, and onset of symptoms between 28 days and 3 months of age. The primary endpoint, achievement of sitting position, was met by 12 (29%) infants in contrast with the natural history of SMA type I, where this milestone was never achieved. Event-free survival, defined as being alive without the use of permanent ventilation, was met in 85% patients at month 12. Treatment with risdiplam over 24 months resulted in further improvement in motor function and developmental milestones [51].

SUNFISH (NCT02908685) was a two-part multicenter, phase 3, double-blind, randomized, placebo-controlled trial assessing efficacy, safety, tolerability, pharmacokinetics, and pharmacodynamics of risdiplam in participants aged 2–25 years with late-onset (type II or type III) SMA [52, 53]. The primary endpoint, the change from baseline in MFM32 total score at month 12, was found to be significantly different from the placebo group.

Risdiplam treatment was not associated with any drug-related safety findings leading to withdrawal. Risdiplam was also tested in another study, Jewelfish (NCT03032172), in adults and children and infants with SMA previously enrolled in other clinical trials or in those who were previously treated with nusinersen, OA, or olesoxime.

##### Risdiplam: real-world data

The drug was approved by the FDA in August 2020, and in March 2021, the European Commission approved it for the treatment of patients affected by SMA who are older than 2 months of age. More recently, FDA has extended the approval also to infants younger than 2 months. The first available real-world data mainly focus on safety in the patients in early access programs. Efficacy real-world data are not yet available.

##### Risdiplam: new developments

A study currently exploring the combination of risdiplam with myostatin inhibitor will be discussed in a separate section.

## Other therapeutical approaches currently in clinical trials

### Antimyostatin

Myostatin (also known as GDF-8) is a negative regulator of skeletal muscle mass [54]. Following suggestions that inhibiting myostatin signaling may provide therapeutic benefit for patients with muscle atrophy, this approach has been used for treatment of SMA [55]. So far, there are two anti-promyostatin drugs in current clinical trials in combination with nusinersen or risdiplam.

### Apitegromab (SRK-015)

Apitegromab (SRK-015) is an anti-promyostatin monoclonal antibody that specifically binds to proforms of myostatin, inhibiting myostatin activation. Following a phase 1 double-blind, placebo-controlled study assessed safety, pharmacokinetic, pharmacodynamics, and immunogenicity of single and multiple ascending doses of apitegromab [55], a Phase 2 Active Treatment Study was performed to evaluate the efficacy and safety of SRK-015 in patients with later-onset spinal muscular atrophy (TOPAZ NCT03921528). Results of the study show sizable motor function gains after 12 months of treatment and 24 months.

A Phase 3, Double-Blind, Placebo-Controlled Trial (SAPPHIRE NCT05156320) in patients with later-onset spinal muscular atrophy receiving background nusinersen or risdiplam therapy has recently started.

### RO7204239

RO7204239 (NCT05115110) is a recycling and antigen sweeping monoclonal antibody (mAb) administered subcutis that binds to human latent myostatin and thereby blocks its conversion to mature myostatin. A two-part, multicenter, randomized, placebo-controlled, double-blind study is currently being performed to investigate safety, tolerability, pharmacokinetics, pharmacodynamics, and efficacy of RO7204239 in combination with risdiplam (RO7034067) in ambulant patients with spinal muscular atrophy.

### Other combination therapies

Following commercial availability and different labels or funding policies in different countries, there are several anecdotal cases of individuals who combined drugs or who added a new treatment after receiving onasemnogene abeparvovec. The possible efficacy or safety profiles of combination therapies have not been reported, and only recently, a few clinical trials have been planned to address this issue.

### Nusinersen following onasemnogene abeparvovec

RESPOND (NCT04089566) is an open-label, single-arm, interventional study that will evaluate the efficacy and safety of nusinersen in participants who previously received IV onasemnogene abeparvovec from at least 2 months. The primary objective of the study is to assess the clinical outcomes using the total HINE Sect. 2 motor milestone score. The secondary objective is to evaluate the safety, tolerability, and additional clinical outcomes.

## New approaches

### Neuromuscular junction

Fatigability defined as a decrease in performance over a given time is frequently reported by SMA patients and their families, in addition to muscle weakness. A possible involvement of the neuromuscular junction has been confirmed by repetitive nerve stimulation showing an abnormal decremental response in SMA patients [56, 57].

A possible involvement of the neuromuscular junction has been further confirmed by clinical studies showing signs of fatigue on consecutive minutes on the 6MWT [58, 59] that in a study were associated with a parallel decremental response on repetitive nerve stimulation [57].

The finding of postsynaptic dysfunction of the neuromuscular junction in SMA suggests that patients may benefit from drugs that facilitate neuromuscular transmission.

### Salbutamol/albuterol

Salbutamol (albuterol in US), a β2-adrenoreceptor agonist, is used in many centers. Following an open-label pilot study and a subsequent study showing an increase in strength [60], salbutamol has been used as an off-label therapy and patients report less fatigability and an increase in endurance [60, 61]. Another paper reported a concordant improvement of 6MWT and neurophysiology following introduction of salbutamol in SMA patients suggests a potential role on neuromuscular junction [62]. No systematic placebo controlled study has however been performed, and in the recent care recommendations, there was no consensus on its use among experts [63, 64].

### Pyridostigmine

Pyridostigmine, an acetylcholinesterase inhibitor, an FDA- and EMA-approved treatment of myasthenia gravis, has been proposed for clinical trials aimed at investigating its efficacy on motor function and fatigability [65].

## New frontiers: treatment of presymptomatic patients and neonatal screening

### Clinical trials in presymptomatic patients

All the three therapeutic approaches have also been used in presymptomatic patients.

The first study to be completed was NURTURE (NCT02386553) using nusinersen in a phase 2, open-label, single-arm, multinational study to evaluate the long-term safety and efficacy of intrathecal nusinersen in infants who initiate treatment before the onset of clinical signs of SMA [66]. All participants achieved the ability to sit without support, 23/25 (92%) achieved walking with assistance, and 22/25 (88%) achieved walking independently, and most importantly, motor milestones were achieved in timelines consistent with normal development in typically developing infants.

Two recent studies report the use of onasemnogene abeparvovec in presymptomatic patients [67, 68]. The study SPR1NT (NCT03505099) was a phase III multicenter, single-arm trial, assessing efficacy and safety of onasemnogene abeparvovec in presymptomatic SMA infants treated within six weeks of age. The results were reported separately for infants with 2 and 3 copies of the SMN2 genes. All patients in both subgroups survived, and none required nutritional or respiratory support.

All infants with 2 SMN2 copies sat independently for ≥ 30 s, which was the primary measure for this subgroup. All children with 3 SMN2 copies stood independently before 24 months and 14 walked independently.

The preliminary results of an ongoing study using risdiplam in presymptomatic patients, Rainbowfish (NCT03779334), indicate a similar trend to those observed when using the other drugs.

## Discussion

The field of SMA has been completely changed by the advent of the new drugs. This is most strikingly evident in type I SMA because of the dramatic changes in survival and improved motor, bulbar, and respiratory function, but it is also obvious in the other forms of SMA as a reduction of the progression of the disease invariably observed in the past in untreated patients.

The new therapies have highlighted a number of issues and challenges. One of the main topics of discussion at the moment is the definition of the new phenotypes. Infants with early onset that are treated in the first months achieve the ability to sit but also develop scoliosis, kyphosis, and other aspects that were not observed as part of the history of type I SMA at the time when infants did not achieve sitting or survived. As these are relatively new findings, no consensus has yet been reached on the best way to address them and to revise the standards of care that have been published just before the new therapies became available [63, 64]. The same applies for older patients. Severe scoliosis requiring surgery was an invariable finding in type 2 SMA and in type III who lost ambulation [64], and there is no information on whether the new therapies will delay the onset and the progression of the curvature.

This also raises the issue of the suitability of the existing clinical tools to measure changes in treated patients. All the available tools were developed to assess the levels of function observed in untreated patients and are not always appropriate to see the positive changes observed after treatment.

There has recently also been an effort to better capture nonmotor functional changes. We recently developed the OrSAT (Oral and Swallowing Abilities Tool), a test specifically developed for recording structured information on various aspects of oral, swallowing, and feeding abilities that can be used since the first months after birth in type I SMA patients [69]. The new tool has been used both to show the progressive decline in a cohort of untreated type I SMA patients and in treated patients providing some information on the changes observed in response to the new therapies [70].

Further studies will also better characterize changes in respiratory function. Both clinical studies and real-world data suggest that infants and children who have no need for respiratory support at the time of treatment often do not develop further need for further support [33, 71, 72]. It will also be of interest how the new therapies will impact the decline in FVC previously invariably observed in untreated patients [73].

Increasing attention has also been paid to the identification of objective nonclinical biomarkers. Neurophysiology, such as the ulnar nerve compound motor action potential amplitude, and other biomarkers in blood or CNS, such as neurofilament levels or SMN protein, may provide additional prognostic information and help to predict response to a medication [74]. Our experience is that muscle imaging studies, such as muscle MRI, providing information on muscle atrophy and replacement may also help to identify patients with more preserved muscles that may better respond to treatment [75].

The ultimate challenge however is the need for neonatal screening. This is becoming increasingly available in many USA states, while in Europe and the rest of the world is more limited [76]. Considering the results of the studies in presymptomatic studies, the early identification of the infants with SMN1 mutations with 2 or 3 SMN2 copies, as included in the clinical trials, would allow to benefit from early treatment with a very high possibility to develop, especially for the infants with 3 SMN2 copies, milestones at the age of their typically developing peers without SMN mutations.

In conclusion, clinical trials and real-world data strongly support the efficacy and safety profiles of the available drugs. The availability of three different options is often challenging for families and clinicians. At the moment, there is not enough published evidence about the superiority of one product compared to the others. There are still a number of important questions that need to be addressed, such as which treatment to initiate, what dosage to use, how to study treatment combinations, and how to study new treatments in the setting of existing effective treatments. Recent ongoing studies, including different dosages or modalities of administration and combination therapies, will be helpful to better understand the pros and cons of each treatment and guide families and clinicians. Further help may also come from the application of new statistical and Artificial Intelligence (AI) methods to real-world data that may allow a better interpretation of the results and the development of prognostic algorithms [77].
